# IL-4 Amplifies the Pro-Inflammatory Effect of Adenosine in Human Mast Cells by Changing Expression Levels of Adenosine Receptors

**DOI:** 10.1371/journal.pone.0024947

**Published:** 2011-09-26

**Authors:** Xiaoyang Hua, Kelly D. Chason, Janki Y. Patel, Warren C. Naselsky, Stephen L. Tilley

**Affiliations:** 1 Department of Medicine, Division of Pulmonary and Critical Care Medicine, and the Center of Environmental Medicine, Asthma and Lung Biology, University of North Carolina at Chapel Hill, Chapel Hill, North Carolina, United States of America; 2 Department of Otolaryngology, Tongji Hospital, Huazhong University of Science and Technology, Wuhan, China; University of Colorado Denver, United States of America

## Abstract

Adenosine inhalation produces immediate bronchoconstriction in asthmatics but not in normal subjects. The bronchospastic effect of adenosine is largely mediated through adenosine-induced mast cell activation, the mechanism of which is poorly understood due to limitations in culturing human primary mast cells. Here, we show that human umbilical cord blood -derived mast cells incubated with the Th2 cytokine IL-4 develop increased sensitivity to adenosine. Potentiation of anti-IgE- induced and calcium ionophore/PMA-induced degranulation was augmented in mast cells cultured with IL-4, and this effect was reduced or abolished by pre-treatment with A_2B_siRNA and selective A_2B_ receptor antagonists, respectively. IL-4 incubation resulted in the increased expression of A_2B_ and reduced expression of A_2A_ adenosine receptors on human mast cells. These results suggest that Th2 cytokines in the asthmatic lung may alter adenosine receptor expression on airway mast cells to promote increased responsiveness to adenosine.

## Introduction

Adenosine is an important modulator of inflammation that is believed to contribute to the pathogenesis of several chronic diseases, including asthma. In asthmatics, adenosine inhalation can elicit immediate bronchoconstriction [1].This effect on the airway can largely be eliminated by pretreatment with mast cell membrane stabilizers and H1 receptor antagonists, suggesting that the bronchoconstriction following adenosine challenge occurs indirectly through activation of mast cells [2], [3].

Adenosine exerts its actions by activating four distinct G-protein coupled receptors, termed A_1_, A_2A_, A_2B_ and A_3_, each with unique tissue distributions, ligand affinities, and intracellular signaling pathways. Mast cells from most species express A_2A_, A_2B_ and A_3_ adenosine receptors [4]. The receptor(s) mediating adenosine-induced mast cell activation has been controversial, which is partially due to high heterogeneity of mast cells. In murine mast cells, *in vitro* and *in vivo* studies have shown that adenosine can both directly activate mast cells and potentiate antigen-induced mast cell activation via the A_3_ adenosine receptor [5], [6], [7], [8]. In the BR-1 mast cell line derived from a canine mastocytoma, adenosine analogue NECA-induced degranulation is blocked by enprophylline, a selective A_2B_ antagonist, suggesting that the A_2B_ receptor mediates the pro-inflammatory effects of adenosine in this mast cell model [9]. Unfortunately, the BR-1 cell line has many features suggesting that it is intrinsically different from human mast cells. For example, BR-1 cells lack FcεRI; they also express A_1_ receptors which are not expressed on mast cells from other species including humans [9]. As such, it is still controversial whether or not investigations using BR-1 cells reflect adenosine signaling pathways in human mast cells.

Human mast cells have been considerably more difficult to obtain and culture. To date, the human mast cell models that have been used for the investigation of adenosine biology include: HMC-1 cell lines, human lung fragments, dispersed human lung mast cells, mast cells isolated from human bronchoalveolar lavage (BAL) fluid, and cultured human umbilical cord blood derived mast cells (HUCBMCs) [4], [10], [11], [12], [13], [14], [15]. HMC-1 is a poorly differentiated malignant mast cell line obtained from a patient with mast cell leukemia. Similar to the BR-1 cell line from dog mastocytoma, the A_2B_ adenosine receptor has been implicated in mediating the pro-inflammatory effects of adenosine in HMC-1 cells [11]. HMC-1 cells also lack several features of human mast cells including the expression of FcεRI [16]. The cell membrane ion channel profile in HMC-1 cells is also unique among mast cell lines studied [17]. Thus, it remains unclear whether or not characteristics of adenosine biology found in HMC-1 cells are applicable to normal human mast cells or mast cells from asthmatics. Human lung fragments, dispersed human lung mast cells, and mast cells isolated from human BAL fluid have also been used to study of adenosine biology. While these studies have revealed interesting data, concerns have been raised in terms of data interpretation because of the poor cell purity and the enzymatic damage to cell integrity during cell preparation.

HUCBMCs are primary cultures from human umbilical cord blood mast cell progenitors [18]. Through *in vitro* differentiation using cytokines, these cells recapitulate many features of mature human mast cells including the expression of FcεRI [19]. Two adenosine studies using HUCBMCs have been reported; however, their conclusions are contradictory. Suzuki et al. showed a robust, dose-dependent inhibitory effect of adenosine on anti-IgE-induced degranulation in cultured HUCBMCs, mediated by A_2A_ receptors [20]. In contrast, a study recently reported by Yip et al. showed a biphasic effect of adenosine in HUCBMCs, implicating the A_1_ receptor in potentiation, and A_2B_ receptor in the inhibition of anti-IgE-induced degranulation [21].

The observation that adenosine-induced bronchoconstriction occurs in asthmatics but not in normal subjects suggests that pro-inflammatory signaling pathways by adenosine may be different in asthmatic mast cells. Since isolating sufficient numbers of mature mast cells from the lungs of asthmatics is not possible for adequate investigation of adenosine receptor biology, and since primary mast cell cultures from asthmatics are extremely hard to establish, we reasoned that incubation of HUCBMCs with IL-4, a cytokine critical in the development of asthma and allergy, may provide an *in vitro* condition more reflective of the mast cell microenvironment in the asthmatic lungs.

## Materials and Methods

### Cell Extraction and Culture

Heparinized cord blood was obtained from the Carolinas Cord Blood Bank at UNC Hospitals; samples were units with volumes or cell counts too low for use by the Cord Blood Bank, and their use for this research was determined to be exempt from the approval by the Biomedical Institutional Review Board at the University of North Carolina at Chapel Hill (UNC-CH). Mononuclear cells (MNCs) were extracted from the blood by density gradient separation (Ficoll-Paque Plus, GE Healthcare). Red blood cells in the MNC fraction were lysed with ACK Lysis Buffer (Quality Biologicals). CD133+ cells were extracted from the MNCs by magnetic bead separation (Miltenyi Biotec). The CD133+ cells were then cultured in Iscove's modified Dulbecco's medium (IMDM; Gibco) containing 100 ng/ml recombinant human SCF (Amgen and Invitrogen), 50 ng/ml recombinant human IL-6 (Amgen and Invitrogen), 1 ng/ml IL-3 (first week only; Invitrogen), 10 mM HEPES, 1 mM L-glutamine, 100 U/ml penicillin, 100 µg/ml streptomycin, 10 µg/ml gentamicin, MEM vitamins (Cellgro), MEM amino acids (Cellgro), 1 mM sodium pyruvate (Cellgro), and 50 µM beta mercaptoethanol (Sigma). For the first six weeks of culture the medium was supplemented with 0.1% BSA (Sigma) and 1% Insulin-Transferine-Selenium (Gibco); afterwards, it was supplemented with 10% FCS (Atlanta Biologicals). Half of the culture medium was changed weekly (2× concentration of SCF and IL-6), and cells were cultured for at least 12 weeks at 1×10^6^ cells/ml. Cells at 12 weeks old were determined to be >95% mast cells by Kimura stain and Tryptase immunofluorescence.

### Immunohistochemical stains of mast cell tryptase

Cells were centrifuged onto poly-l-lysine coated coverslips, fixed in methanol and acetone, and blocked with 50% FBS, 5% BSA, and 0.1% gelatin. Cells were probed with anti-tryptase (Clone G3, MAB1222, Millipore) or isotype control (MABC002, Millipore). Secondary antibody was Rhodamine-conjugated anti-mouse antibody (AP124R, Chemicon). Coverslips were mounted on slides with Vectashield containing DAPI (Vector Laboratories).

### Hexosaminidase Release Assay

Reagents were obtained from Sigma-Aldrich unless otherwise noted. HUCBMCs were incubated for 3 days at 1×10^6^ cells/ml and 37°C with 10 ng/ml recombinant human IL-4 (Invitrogen), 5 µg/ml Human Myeloma IgE (Meridian Life Science), or both in culture medium. After washing twice and resuspending in Siraganian Buffer, 1×10^5^ cells were transferred to wells of 96-well microtiter plates. In experiments using adenosine receptor antagonists VUF 5574, PSB 1115 (Tocris), and MRS 1754, cells were incubated in the presence and absence of antagonists for 30 min prior to adenosine addition. Cells were incubated in the presence and absence of adenosine or the adenosine analogue 2-chloro-N^6^-(3-iodobenzyl)-adenosine-5′-N-methyl-carboxamide (2-Cl-IB-MECA) for 30 min. Cells were then stimulated with 1 µg/ml anti-human IgE, or with 60 nM A23187 and 100 ng/ml Phorbol 12-myristate13-acetate (PMA), at 37°C for 30 min. Reactions were terminated by incubating cells on ice and centrifuging at 350× g for 10 min at 4°C. The extent of mast cell degranulation was determined by comparing levels of hexosaminidase activity in the supernatant and cell pellets. Hexosaminidase activity was determined by incubating supernatant and cell lysate with 1 mMp-nitrophenyl-N-acetyl-B-d-glucosaminidine in citrate buffer (0.1 M citric acid, 0.1 M sodium citrate, pH 4.5) for 1 h at 37°C. The reaction was terminated by adding 0.1 M Na_2_CO_3_/NaHCO_3_, and the absorbance was measured at 405 nm. Hexosaminidase release was expressed as a percentage of the total amount of hexosaminidase present in the cells as described previously [22].

### siRNA knockdown

Cells in antibiotic-free medium were treated with 5 µg/ml human myeloma IgE (Meridian Life Science), 10 ng/ml IL-4 (Invitrogen), and 300 nM A_2B_, A_3_, and Non-Targeting siRNA(ONTARGET plus SMARTpool, A_2B_: L-005417-00; A_3_: L-005418-00; Non-Targeting: D-001810-10, Thermo FisherScientific, Lafayette, CO) using LipofectamineRNAiMAX (Invitrogen) prepared in OptiMEM (Invitrogen). Cells were incubated at 37°C, 5% CO2. After 48 hours, an aliquot was removed for RNA extraction and qPCR. The remaining cells were incubated for another 24 hours. Hexosaminidase release was measured after treatment with adenosine and anti-IgE.

### RNA extraction and Real-Time PCR

Total RNA was extracted from HUCBMCs, incubated with IgE, IL-4 or IgE plus IL-4 as above, with RNA-bee (Tel-Test). RNA was treated with RQ1 DNAse (Promega) and reverse-transcribed using the High-Capacity cDNA Archive Kit (Applied Biosystems). Gene expression relative to controls was determined by Real-Time PCR, using the Universal Master Mix (Applied Biosystems) and Gene Expression Assays (A_1_: Hs00181231_m1; A_2A_: Hs00169123_m1; A_2B_: Hs00386497_m1; A_3_: Hs00181232_m1; GAPDH: Hs99999905_m1, Applied Biosystems) on a 7300 Real Time PCR System (Applied Biosystems). Relative mRNA expression, normalized to human GAPDH mRNA, was calculated by the comparative Ct method.

## Results

### Adenosine potentiates anti-IgE-induced degranulation of HUCBMCs

**Figure 1:**
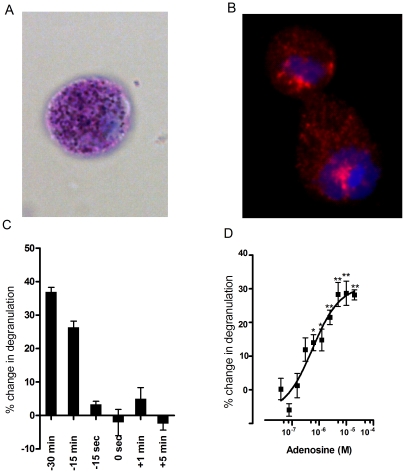
Adenosine potentiates anti-IgE-induced degranulation of human umbilical cord blood derived mast cells. CD133^+^ cells from human umbilical cord blood were isolated by magnetic bead separation and cultured in Iscove's modified Dulbecco's medium (IMDM) supplemented with human SCF, IL-6 and IL-3. Mast cells were identified by Kimura stain (A) and tryptase immunofluorescence (B). Cell purity at 12 weeks was >95%. All cells were then treated with human IL-4 (10 ng/ml) and IgE (5 µg/ml) for three days. Mast cell degranulation was triggered by anti-IgEmAb (1 µg/ml) and evaluated by measuring hexosaminidase release. The time course response of adenosine on anti-IgE-induced mast cell degranulation was examined by the addition of adenosine (200 µM in PBS) at the indicated time points (C). The dose response of adenosine on anti-IgE-induced mast cell degranulation was tested by adding adenosine at indicated concentration 30 min prior to anti-IgE challenge (D). Data are presented as the mean % change in hexosamindase release by adenosine over the PBS treated cells, ± SEM. *p<0.05, ** p<0.01 vs. PBS treated cells by student *t* test.

HUCBMCs were cultured for 12 weeks. Cell purity was greater than 95% after 12 weeks in culture as determined by Kimura and tryptase immunohistochemical stains (Figure 1A and 1B). Prior to examining the effects of adenosine, we treated cultured human mast cells with IgE antibodies and IL-4 for 3 days. Adenosine failed to directly degranulate HUCBMCs (data not shown); instead, adenosine enhanced the FcεRI-induced mast cell degranulation. Figure 1C shows how the timing of adenosine exposure influences anti-IgE-induced mast cell degranulation. Adenosine (200 µM) added 15 and 30 min prior to the antigen challenge significantly increased anti-IgE-induced mast cell degranulation. When adenosine was added 15 s prior to, or 1 and 5 min after antigen challenge, no potentiation was observed. These findings indicate that adenosine added 15–30 min prior to antigen challenge can elicit a potentiating effect on anti-IgE-induced degranulation of HUCBMCs.

The dose response relationship of adenosine on anti-IgE-induced degranulation of HUCBMCs is shown in Figure 1D. The minimum concentration of adenosine with a reproducible potentiating effect was 6.25 µM (14%±2.4% increase) (P<0.05 vs. PBS treated cells), and the maximal potentiating effect was achieved at 100 µM (28.7%±3.6% increase). Collectively, these data demonstrate that adenosine pretreatment is capable of potentiating anti-IgE-induced degranulation of HUCBMCs.

### Effect of A_3_ agonistsand antagonists

**Figure 2:**
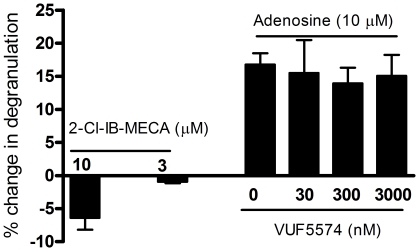
Effect of A_3_ agonist and antagonist on anti-IgE-triggered mast cell degranulation. HUCBMCs were cultured for 12 weeks and treated with IL-4 and IgE for 3 days. A_3_ selective agonist 2-Cl-IB-MECA at 3 and 10 µM was added 30 min prior to anti-IgE challenge. Mast cell degranulation was then quantitated by measuring hexosaminidase release from cells. Adenosine (10 µM) was used as a positive control. The effect of the A_3_ receptor antagonist VUF5574 on adenosine-induced enhancement of anti-IgE triggered mast cell degranulation was also tested. IL-4/IgE treated HUCBMCs were incubated with VUF5574 at indicated concentrations for 30 min. The cells were then treated with adenosine (10 µM) for 30 min. Mast cell degranulation was triggered by the addition of anti-IgE. Data are from 3–6 different lines of cells and are presented as the mean % change in hexosaminidase release over the vehicle treated cells ± SEM. *p<0.05 vs. adenosine treated cells, by paired student *t* test.

Previous studies with murine mast cells have indicated that the potentiating effects of adenosine are mediated by A_3_ adenosine receptors [6]. In order to determine the adenosine receptor subtype responsible for the potentiating effect of adenosine on HUCBMCs, we treated cells with the A_3_ adenosine receptor selective agonist 2-Cl-IB-MECA prior to activation with anti-IgE. As shown in Figure 2, pretreatment with 2-Cl-IB-MECA at 3 and 10 µM failed to potentiate anti-IgE-induced mast cell degranulation. As a positive control, we still observed a potentiating effect by adenosine at 10 µM in the same experiments.

In addition, we blocked A_3_ adenosine receptors by VUF5574, a highly selective A_3_ adenosine receptor antagonist, and then tested the capacity of adenosine to potentiate anti-IgE-induced degranulation. The potentiating effect of adenosine was consistently observed in cells pretreated with VUF5574 ranging from 30 to 3000 nM (Figure 2). These data with an A_3_-selective agonist and A_3_-selective antagonist indicate that the potentiating effect of adenosine on HUCBMCs is not mediated by A_3_ adenosine receptors.

### A_2B_ antagonists block adenosine's capacity to potentiate degranulation of HUCBMCs

**Figure 3:**
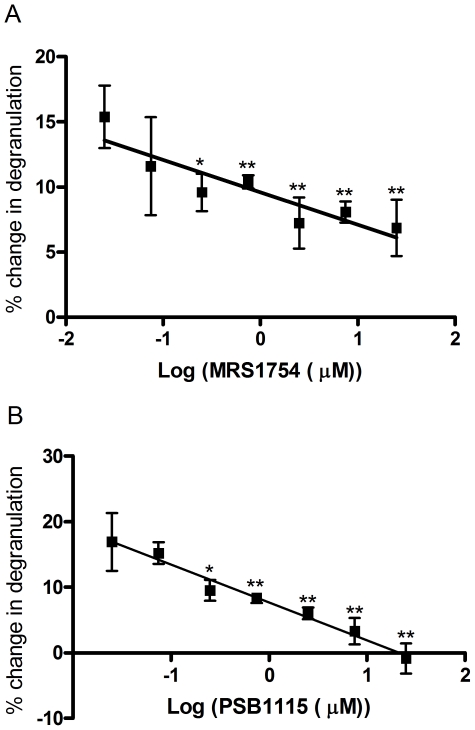
Adenosine-induced potentiation of degranulation is abolished by A_2B_ antagonism. IL-4/IgE treated HUCBMCs were incubated with the A_2B_ receptor antagonists MRS1754 (A) and PSB1115 (B) at indicated concentration for 30 min. Cells were then treated with adenosine (10 µM) or PBS for 30 min. Mast cell degranulation was stimulated by the addition of anti-IgE. Data are from 3–4 different lines of cells and are presented as the mean % change in hexosaminidase release over the vehicle treated cells ± SEM. *p<0.05; **p<0.01 vs. PBS treated cells, by student *t* test.

We next tested the capacity of A_2B_ antagonists to block the potentiating effects of adenosine on anti-IgE-induced degranulation of HUCBMCs. As shown in Figure 3A, the A_2B_ adenosine receptor antagonist MRS1754 from 0–25 µM dose-dependently reduced the potentiating effect of adenosine on anti-IgE-induced degranulation. The minimal effective concentration of MRS1754 was 75 nM (p<0.01 vs. control cells). With the MRS1754 concentration increased to 25 µM, the potentiating effect of adenosine was significantly inhibited (the potentiating effect of adenosine at 25 µM was 6.9%±2.2% vs. 21%±2.2% in control cells, p<0.01). In addition, we also tested the effect of a second A_2B_ selective antagonist PSB1115. As shown in Figure 3B, PSB1115, at concentrations ranging from 0–25 µM, significantly decreased the potentiating effect of adenosine on anti-IgE-induced mast cell degranulation. Compared with MRS1754, PSB1115 showed greater efficacy, as PSB1115 at 25 µM completely abolished the capacity of adenosine to potentiate anti-IgE-induced degranulation. The minimal effective concentration of PSB1115 was also 75 nM, a concentration that is highly selective for the A_2B_ adenosine receptor based on the published Ki value for this compound. Collectively, these data indicate that the potentiating effect of adenosine on anti-IgE-induced degranulation of HUCBMCs is mediated by A_2B_ adenosine receptors.

### A_2B_ but not A_3_ siRNA inhibits potentiation of mast cell degranulation by adenosine

**Figure 4:**
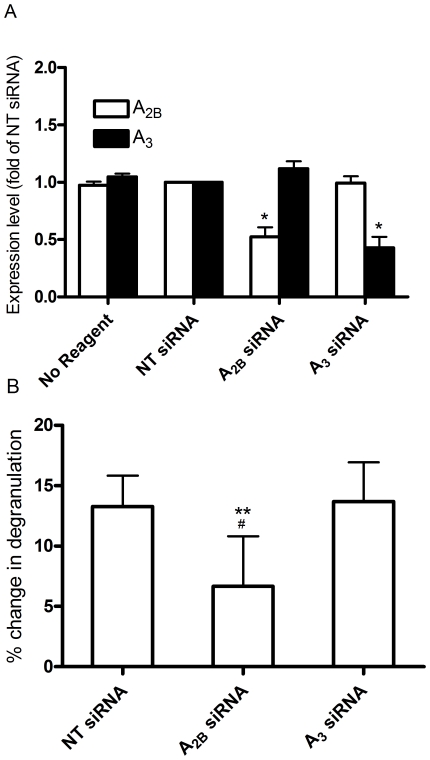
A_2B_ siRNA attenuates adenosine-induced potentiation ofanti-IgE-triggered mast cell degranulation. A. Expression levels of A_2B_ and A_3_ adenosine receptors determined by realtime PCR after pretreatment with A_2B_ and A_3_siRNA. NT siRNA = non-targeting siRNA. Data are from 6different lines of cells and expressed as fold of NT siRNA treated cells. * p<0.05 vs. NT siRNA treated cells. B. Adenosine-induced potentiation of anti-IgE-triggered degranulation of HUCBMCs treated with NT, A_2B_ and A_3_siRNA. IgE/IL-4 treated HUCBMCs were incubated with NT, A_2B_ and A_3_siRNA for 72 h, respectively. These cells were then treated with adenosine (10 µM) or PBS for 30 min. Hexosaminidase release was triggered by the addition of anti-IgE. Data are from 6 different lines of cells and are presented as the mean % change in hexosaminidase release over the vehicle treated cells ± SEM. **p<0.01 vs. A_3_ siRNA treated cells; #p = 0.049, vs. NT siRNA treated cells by paired student *t* test.

In order to further demonstrate that A_2B_ adenosine receptors mediate adenosine-induced potentiation of mast cell degranulation by anti-IgE, we used siRNA to knock down the A_2B_ receptors in HUCBMCs. As shown in Figure 4A, pretreatment with siRNA targeting human A_2B_ receptors reduced A_2B_ transcript levels by approximately 50%. Likewise, siRNA targeting A_3_ receptors also reduced A_3_ expression by approximately 50%. Adenosine induced potentiation of anti-IgE induced mast cell degranulation was significantly reduced by A_2B_ but not A_3_siRNA (Figure 4B). Collectively, these data and our pharmacological experiments with selective adenosine receptor ligands indicate that A_2B_ receptors mediate the potentiation of anti-IgE-induced degranulation by adenosine in human mast cells.

### Adenosine effects on IgE and IL-4 treated and non-treated cells

**Figure 5:**
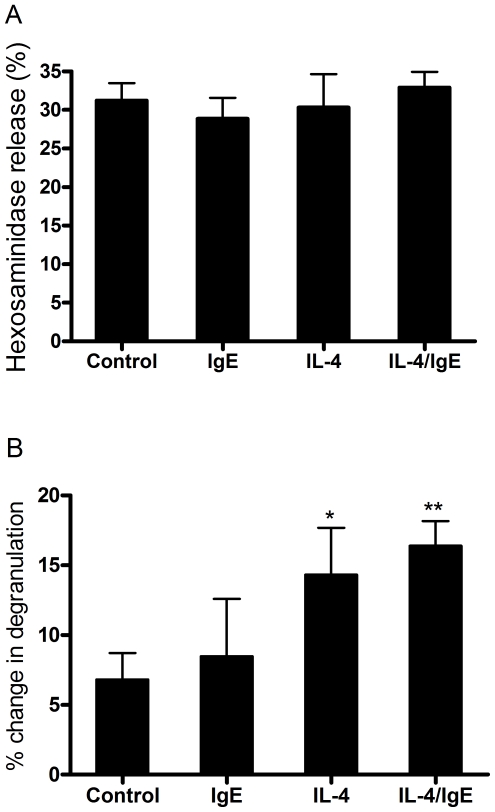
Potentiation of degranulation by adenosine in IL-4, IgE and IL-4/IgE treated and non-treated HUCBMCs. A. IL-4/IgE treated and non-treated HUCBMCs were incubated with A23187 (60 nM) plus PMA (100 ng/ml) for 30 min. Mast cell degranulation was then evaluated by measuring hexosaminidase release. Data are from 5 different lines of cells. B. IL-4, IgE and IL-4/IgE-treated and non-treated HUCBMCs were incubated with adenosine (200 µM) for 30 min followed by the addition of A23187 (60 nM) plus PMA (100 ng/ml) to trigger mast cell degranulation. Data are from 5 different cell lines and are presented as the mean % change in hexosaminidase release over the vehicle treated cells ± SEM. ** P<0.01; * P<0.05 vs. controls, by paired student *t* test.

Previous studies have shown that adenosine induces bronchoconstriction in asthmatics but not healthy controls, and that adenosine-induced bronchoconstriction can be largely inhibited by suppressing mast cells [1], [2], [3]. Thus, it has long been posited that the Th2 cytokines in asthmatic patients change the adenosine signaling in mast cells and confer adenosine the ability to produce bronchospasm. In order to test this hypothesis, we compared the effect of adenosine on the degranulation of naïve vs. IL-4/IgE treated HUCBMCs. In this study, we used A23187 plus PMA instead of anti-IgE to directly induce mast cell degranulation to exclude the confounding effects of IL-4 and IgE-induced up-regulation of FcεRI expression [19]. As shown in Figure 5A, A23187 plus PMA induced similar degranulation in both naïve and IL-4/IgE treated mast cells (p = 0.92, n = 5). Next, we treated both naïve and IL-4/IgE pre-incubated mast cells with adenosine for 30 min followed by the addition of A23187 plus PMA. As shown in Figure 5B, in cells not incubated with IL-4, adenosine modestly increased degranulation by 6.8%±1.9%, whereas in IgE, IL-4, and IgE plus IL-4 treated cells, adenosine increased degranulation by 8.5%±4.1% (P>0.05), 14.3%±3.4% (P<0.05) and 16.4%±1.8% (P<0.05) respectively. These data indicate that IL-4 can enhance the pro-inflammatory effect of adenosine on mast cells.

### Expression levels of adenosine receptors on human mast cells after IL4 and IgE treatment

**Figure 6:**
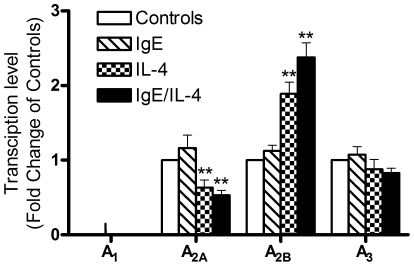
Expression levels of adenosine receptors in IL-4, IgE and IL-4/IgE treated and non-treated HUCBMCs. HUCBMCs were treated with vehicle, IL-4, IgE and IL-4/IgEfor 72 h, respectively. Total RNA was extracted from these cells and adenosine receptor expression levels were determined by real time PCR. Data are from 6–11 different lines of cells and are presented as fold change from non-treated cells ± SEM. **P<0.01 by student *t* test.

One possible explanation for the enhanced response to adenosine in IL-4 pretreated human mast cells is that IL-4 may alter the expression levels of adenosine receptors mediating the effect of adenosine. In order to test this hypothesis, we examined the effect of IL-4, IgE, and IL-4/IgE pre-treatment on adenosine receptor expression in HUCBMCs. As shown in Figure 6, expression levels of the A_2B_ receptor increased 2.4 fold in IL-4/IgE-treated cells (p<0.001), 1.9 fold in IL-4 treated cells (p<0.001), but were unchanged in IgE treated cells (p = 0.56). In addition, pretreatment with both IL-4 and IgE/IL-4 significantly reduced the expression levels of A_2A_ receptors. Expression levels of the A_2A_ receptors were decreased by 37% in IL-4/IgE treated cells ((p<0.001) and 48% (P<0.01) in IL-4 treated cells, but were unchanged in IgE treated cells (P = 0.2). These data suggest that IL-4 enhances the pro-inflammatory effects of adenosine in mast cells by up-regulating A_2B_ receptors and down-regulating A_2A_ receptors.

## Discussion

Inhalation of 5′AMP, which is rapidly metabolized to adenosine by ecto-nucelotidases on airway epithelia, produces immediate bronchoconstriction in asthmatics but not in normal individuals. Endogenous adenosine levels in BAL and exhaled breath condensates from asthmatics are elevated [23], [24]. These findings suggest a pro-inflammatory role for adenosine in asthma pathogenesis. Previous *in vivo* human studies have indicated that mast cells are one of the major intermediaries in adenosine-induced bronchoconstriction; however, the underlying mechanism is yet to be fully understood, which is partially due to the difficulty in obtaining and culturing human mast cells. We and others have conducted a number of animal studies both *in vitro* and *in vivo* in order to determine the precise mechanism of adenosine-induced bronchoconstriction. While specific adenosine receptors have been implicated by these studies, it remains unclear whether or not these observations are reflective of adenosine biology in human mast cells.

In this study, human umbilical cord blood derived mast cells (HUCBMCs) were cultured *in vitro* and the role of adenosine and its receptors during mast cell degranulation was investigated. After 12 weeks of *in vitro* differentiation, these mast cells were incubated with IL-4 and IgE. Since adenosine induces bronchoconstriction via mast cell activation only in asthmatics, we posited that the Th2 microenvironment might be critical to adenosine's capacity to activate the mast cell in the asthmatic lung. Our experiments revealed that when incubated with IL-4/IgE, adenosine potentiated anti-IgE-induced degranulation of HUCBMCs, and antagonism of A_2B_ but not A_3_ receptors abolished this pro-inflammatory effect of adenosine. siRNA targeting A_2B_ but not A_3_ receptors also attenuated adenosine-induced potentiation of mast cell degranulation. In naïve mast cells that were not exposed to IL-4 and IgE, a very modest potentiating effect on mast cell degranulation was seen; however, this pro-inflammatory signal was significantly increased in the presence of IL-4, IgE/IL-4 but not IgE. Pre-treatment with IL-4 or IgE plus IL-4 significantly up-regulated A_2B_ and down-regulated A_2A_ receptor expression in mast cells, providing a plausible mechanism for the enhanced potentiation that we observed.

Our findings of a potentiating effect by adenosine in HUCBMCs are similar to findings with human lung mast cells. Peachell et al. found that adenosine at 1 µM potentiated antigen-induced degranulation of both dispersed and purified human lung mast cells [15]. Hughes et al. reported adenosine at concentrations ranging from 0.1 to 100 µM potentiated degranulation of mechanically dispersed human lung mast cells if added prior to immunological challenge [13]. Since the isolated mast cells in these studies were not from asthmatic patients, these data suggested that adenosine might elicit pro-inflammatory effects in non-asthmatics. This concept was further supported by a study using human BAL mast cells. Forsythe et al. demonstrated that adenosine can also induce degranulation of BAL mast cells from normal subjects [10]. These data indicate that adenosine may still induce mast cell activation in normal people, but the extent of mast cell degranulation by adenosine may not be of sufficient magnitude to elicit bronchoconstriction.

Two previous studies have investigated the effect of adenosine on HUCBMCs. Suzuki et al. reported a dose-dependent inhibitory effect of adenosine on anti-IgE-induced degranulation of cultured HUCBMCs [20]. Since this inhibition by adenosine was abolished by the A_2A_ selective antagonist ZM241385, this group concluded that A_2A_ adenosine receptors were responsible for these inhibitory effects. Yip et al. reported a biphasic effect of adenosine on anti-IgE-induced degranulation in HUCBMCs [21]. Adenosine potentiated anti-IgE-induced degranulation at lower concentrations (10^−9^to10^−7^ M), whereas at higher concentration (more than 10^−7^ M), adenosine inhibited anti-IgE-induced degranulation. In addition, since the potentiating effect of adenosine in this study could only be reproduced by an A_1_ but not A_3_ adenosine receptor agonist, and the inhibitory effect of adenosine could not be reproduced by an A_2A_ agonist, this group concluded that the adenosine receptors responsible for the potentiating and inhibitory effect of adenosine were A_1_ and A_2B_, respectively. There are several potential explanations for these divergent results concerning the effects of adenosine on degranulation of HUCBMCs. First, in the study by Suzuki et al., adenosine and other nucleotides were routinely supplemented at high concentrations in the culture media.This chronic exposure to adenosine might have changed the properties of the adenosine receptors on mast cells and influenced the effects of adenosine added during the experiments. Second, the purity of CD34+ mast cell progenitors in the negatively selected mixture from the buffy coat preparation was not determined in the report by Yip et al.; therefore, it is unknown what percentage of cultured cells were actually differentiated from mast cell progenitors [25]. Third, neither study investigated the effect of adenosine on human mast cells in the presence of Th2 cytokines. To the best of our knowledge, our study is the first to investigate the effects of adenosine on mast cell degranulation in primary cultures of human mast cells after pre-treatment with IL-4. The data presented here suggests that the capacity of adenosine to elicit bronchoconstriction in asthmatic, but not normal subjects, could be a consequence of altered adenosine receptor expression on mast cells exposed to the Th2 environment in the asthmatic lung.

The adenosine receptor responsible for the pro-inflammatory effects of adenosine in mast cells has been controversial. Experiments using murine mast cells have shown that adenosine, acting through A_3_ adenosine receptors, directly degranulates mast cells *in vivo* and enhances antigen-induced degranulation *in vitro* [5], [6], [7], [8]. This capacity of adenosine to activate mast cells is believed to contribute to the development of many features of asthma including edema, airway hyperresponsiveness, airway obstruction and inflammatory cell infiltration [5], [7], [26], [27]. Although A_3_ receptors are up-regulated in asthmatic lungs, data supporting a pro-inflammatory role for this receptor in humans is distinctly lacking. To the contrary, stimulation of A_3_ receptors has been shown to inhibit activation of human eosinophils [28].

In addition to A_3_ receptors, mast cells express A_2B_ adenosine receptors. However, the modulatory effect of A_2B_ adenosine receptors on inflammation is also incompletely understood. In murine mast cells, our group has previously reported that mast cells lacking A_2B_ receptors exhibited an exaggerated response to antigen [29], suggesting an anti-inflammatory role forA_2B_ adenosine receptors in mice. An anti-inflammatory role for the A_2B_ receptor on immune cells has been suggested by several other groups. For instance, activation of A_2B_ receptors have been reported to reduce the severity of inflammation in LPS-induced murine macrophage activation, murine sepsis, and a mouse model of ventilation-induced lung injury [30], [31], [32]. In purified and dispersed human lung mast cells, adenosine has been shown to inhibit anti-IgE-induced degranulation at high concentrations [15]. Since this receptor has the lowest affinity for adenosine and can only be activated by high concentrations of adenosine, it is plausible that the A_2B_ receptor might be anti-inflammatory in human mast cells. Further supporting this notion is the inability of the A_2A_ agonist CGS21680 to re-capitulate the inhibitory effects of high adenosine concentrations on anti-IgE-induced degranulation of HUCBMCs [21]. However, there have also been several studies showing a pro-inflammatory effect of A_2B_ receptors in both humans and mice. For example, Ryzhov et al. have shown that adenosine exerts a pro-inflammatory effect via A_2B_ receptors in murine mast cells and murine macrophages [33], [34]. In addition, this group has shown that the non-selective adenosine analogue NECA causes release of IL-8, IL-4 and IL-13 by the HMC-1 mast cell line, and that these effects are abolished by A_2B_ receptor antagonists [11], [35]. While these findings have been widely extrapolated to suggest that similar pathways are present in asthmatics, direct evidence supporting a pro-inflammatory role for A_2B_ on normal or asthmatic human mast cells has not been previously demonstrated.

A plausible explanation addressing the discordant reports above has been lacking; however, several observations may partially explain some of these discrepancies about the role of adenosine receptors on mast cells. First, adenosine evokes mast cell degranulation when added to BAL preparations containing mast cells and other immune cells, but not purified or dispersed lung mast cells, suggesting the possibility of an indirect effect of adenosine on mast cells through paracrine signals released by co-existing inflammatory cells in BAL following activation by adenosine [10]. As such, the crosstalk between mast cells and other immune cells after the activation of adenosine signaling pathways may be critical *in vivo*, and therefore difficult to elucidate using *in vitro* methods. Second, a recent study suggested that adenosine receptors might have the capacity to change their modulatory effects on inflammation in *vivo* under different inflammatory conditions [36]. Third, there are species differences regarding adenosine receptor functions on mast cells between humans and mice. Fourth, mast cells are highly heterogeneous, and different inflammatory environments, different culture and differentiating conditions in individual studies may change the property of these cells *in vitro* [37]. Lastly, adenosine has different effects on acute vs. chronic inflammation [38]. Given these complexities of adenosine receptor signaling, further investigation with selective adenosine receptor ligands in humans *in vivo* may be necessary to truly reveal their therapeutic potential as well as adverse effects.

We found that IL-4 could significantly increase the expression levels of A_2B_ receptors, and that this change in expression was accompanied by an enhanced pro-inflammatory effect of adenosine on human mast cells. These data support a pro-inflammatory role for the A_2B_ receptor on human mast cells following acute exposure to adenosine, and also suggest that the bronchospastic effect of adenosine in asthmatics might be a result of increased A_2B_ receptor expression on mast cells in asthmatics. We also found that the IL-4 treated human mast cells had decreased A_2A_ receptor expression. A_2A_ adenosine receptors have consistently been shown to transmit anti-inflammatory signals to immune cells, including mast cells [20], [39], [40]. These observations of changes in adenosine receptor expression following IL-4 incubation differ from a previously published study by Versluis et al. with HMC-1 cells incubated with IL4/IL-13 in several ways [41]. First, we used primary cultures of human mast cell rather than the HMC-1 cell line, which lacks many cardinal features of mast cells important in the pathogenesis of asthma (e.g. FcεRI and different cell membrane ion channel profiles). Second, in the study by Versluis et al., only combined treatment with IL-4 and IL-13, but not each cytokine alone, resulted in changes in expression levels of adenosine receptors. Third, the overall conclusion by Versluis was that reduction in A_2A_ expression drove the enhanced responsiveness to adenosine. In contrast, our study shows significant effects of IL-4 on increasing A_2B_ expression and decreasing A_2A_ expression, as well as consistent enhancement of adenosine-induced degranulation in IL-4-treated cells.

Our results showing that IL-4 enhances mast cell responsiveness to adenosine through changes in adenosine receptor expression provides a plausible explanation for the long-standing observation that mast cells in the asthmatic lung are readily activated by adenosine. Changes in adenosine receptor expression as a result of specific cytokines present in the local milieu might explain the capacity of adenosine to act as both a pro- and anti-inflammatory mediator. Further elucidation of specific changes in adenosine receptor expression as a result of inflammatory mediators present in different diseases may identify novel targets for therapy.

## References

[1] Cushley MJ, Tattersfield AE, Holgate ST (1983). Inhaled adenosine and guanosine on airway resistance in normal and asthmatic subjects.. Br J Clin Pharmacol.

[2] Phillips GD, Polosa R, Holgate ST (1989). The effect of histamine-H1 receptor antagonism with terfenadine on concentration-related AMP-induced bronchoconstriction in asthma.. Clin Exp Allergy.

[3] Phillips GD, Scott VL, Richards R, Holgate ST (1989). Effect of nedocromil sodium and sodium cromoglycate against bronchoconstriction induced by inhaled adenosine 5′-monophosphate.. Eur Respir J.

[4] Forsythe P, Ennis M (1999). Adenosine, mast cells and asthma.. Inflamm Res.

[5] Tilley SL, Wagoner VA, Salvatore CA, Jacobson MA, Koller BH (2000). Adenosine and inosine increase cutaneous vasopermeability by activating A(3) receptors on mast cells.. J Clin Invest.

[6] Salvatore CA, Tilley SL, Latour AM, Fletcher DS, Koller BH (2000). Disruption of the A(3) adenosine receptor gene in mice and its effect on stimulated inflammatory cells.. J Biol Chem.

[7] Tilley SL, Tsai M, Williams CM, Wang ZS, Erikson CJ (2003). Identification of A3 receptor- and mast cell-dependent and -independent components of adenosine-mediated airway responsiveness in mice.. J Immunol.

[8] Zhong H, Shlykov SG, Molina JG, Sanborn BM, Jacobson MA (2003). Activation of murine lung mast cells by the adenosine A3 receptor.. J Immunol.

[9] Auchampach JA, Jin X, Wan TC, Caughey GH, Linden J (1997). Canine mast cell adenosine receptors: cloning and expression of the A3 receptor and evidence that degranulation is mediated by the A2B receptor.. Mol Pharmacol.

[10] Forsythe P, McGarvey LP, Heaney LG, MacMahon J, Ennis M (1999). Adenosine induces histamine release from human bronchoalveolar lavage mast cells.. Clin Sci (Lond).

[11] Feoktistov I, Biaggioni I (1995). Adenosine A2b receptors evoke interleukin-8 secretion in human mast cells. An enprofylline-sensitive mechanism with implications for asthma.. J Clin Invest.

[12] Peachell PT, Columbo M, Kagey-Sobotka A, Lichtenstein LM, Marone G (1988). Adenosine potentiates mediator release from human lung mast cells.. Am Rev Respir Dis.

[13] Hughes PJ, Holgate ST, Church MK (1984). Adenosine inhibits and potentiates IgE-dependent histamine release from human lung mast cells by an A2-purinoceptor mediated mechanism.. Biochem Pharmacol.

[14] Church MK, Hiroi J (1987). Inhibition of IgE-dependent histamine release from human dispersed lung mast cells by anti-allergic drugs and salbutamol.. Br J Pharmacol.

[15] Peachell PT, Lichtenstein LM, Schleimer RP (1991). Differential regulation of human basophil and lung mast cell function by adenosine.. J Pharmacol Exp Ther.

[16] Nilsson G, Blom T, Kusche-Gullberg M, Kjellen L, Butterfield JH (1994). Phenotypic characterization of the human mast-cell line HMC-1.. Scand J Immunol.

[17] Bradding P (2005). Mast cell ion channels.. Chem Immunol Allergy.

[18] Theoharides TC, Kempuraj D, Tagen M, Vasiadi M, Cetrulo CL (2006). Human umbilical cord blood-derived mast cells: a unique model for the study of neuro-immuno-endocrine interactions.. Stem Cell Rev.

[19] Yamaguchi M, Sayama K, Yano K, Lantz CS, Noben-Trauth N (1999). IgE enhances Fc epsilon receptor I expression and IgE-dependent release of histamine and lipid mediators from human umbilical cord blood-derived mast cells: synergistic effect of IL-4 and IgE on human mast cell Fc epsilon receptor I expression and mediator release.. J Immunol.

[20] Suzuki H, Takei M, Nakahata T, Fukamachi H (1998). Inhibitory effect of adenosine on degranulation of human cultured mast cells upon cross-linking of Fc epsilon RI.. Biochem Biophys Res Commun.

[21] Yip KH, Wong LL, Lau HY (2009). Adenosine: roles of different receptor subtypes in mediating histamine release from human and rodent mast cells.. Inflamm Res.

[22] Nguyen M, Solle M, Audoly LP, Tilley SL, Stock JL (2002). Receptors and signaling mechanisms required for prostaglandin E2-mediated regulation of mast cell degranulation and IL-6 production.. J Immunol.

[23] Driver AG, Kukoly CA, Ali S, Mustafa SJ (1993). Adenosine in bronchoalveolar lavage fluid in asthma.. Am Rev Respir Dis.

[24] Csoma Z, Huszar E, Vizi E, Vass G, Szabo Z (2005). Adenosine level in exhaled breath increases during exercise-induced bronchoconstriction.. Eur Respir J.

[25] Wang XS, Yip KH, Sam SW, Lau HY (2006). Buffy coat preparation is a convenient source of progenitors for culturing mature human mast cells.. J Immunol Methods.

[26] Hua XCK, Fredholm BB, Deshpande DA, Penn RB, Tilley SL (2008). Adenosine Induces Airway Hyperresponsiveness through Activation of A3 Receptors on Mast Cells.. J Allergy Clin Immunol.

[27] Young HW, Molina JG, Dimina D, Zhong H, Jacobson M (2004). A3 adenosine receptor signaling contributes to airway inflammation and mucus production in adenosine deaminase-deficient mice.. J Immunol.

[28] Knight D, Zheng X, Rocchini C, Jacobson M, Bai T (1997). Adenosine A3 receptor stimulation inhibits migration of human eosinophils.. J Leukoc Biol.

[29] Hua X, Kovarova M, Chason KD, Nguyen M, Koller BH (2007). Enhanced mast cell activation in mice deficient in the A2b adenosine receptor.. J Exp Med.

[30] Eckle T, Grenz A, Laucher S, Eltzschig HK (2008). A2B adenosine receptor signaling attenuates acute lung injury by enhancing alveolar fluid clearance in mice.. J Clin Invest.

[31] Yang D, Zhang Y, Nguyen HG, Koupenova M, Chauhan AK (2006). The A2B adenosine receptor protects against inflammation and excessive vascular adhesion.. J Clin Invest.

[32] Csoka B, Nemeth ZH, Rosenberger P, Eltzschig HK, Spolarics Z A2B adenosine receptors protect against sepsis-induced mortality by dampening excessive inflammation.. J Immunol.

[33] Ryzhov S, Zaynagetdinov R, Goldstein AE, Novitskiy SV, Blackburn MR (2008). Effect of A2B adenosine receptor gene ablation on adenosine-dependent regulation of proinflammatory cytokines.. J Pharmacol Exp Ther.

[34] Ryzhov S, Zaynagetdinov R, Goldstein AE, Novitskiy SV, Dikov MM (2008). Effect of A2B adenosine receptor gene ablation on proinflammatory adenosine signaling in mast cells.. J Immunol.

[35] Feoktistov I, Ryzhov S, Goldstein AE, Biaggioni I (2003). Mast cell-mediated stimulation of angiogenesis: cooperative interaction between A2B and A3 adenosine receptors.. Circ Res.

[36] Cohen MV, Yang X, Downey JM A(2b) adenosine receptors can change their spots.. Br J Pharmacol.

[37] Galli SJ, Kalesnikoff J, Grimbaldeston MA, Piliponsky AM, Williams CM (2005). Mast cells as “tunable” effector and immunoregulatory cells: recent advances.. Annu Rev Immunol.

[38] Zhou Y, Mohsenin A, Morschl E, Young HW, Molina JG (2009). Enhanced airway inflammation and remodeling in adenosine deaminase-deficient mice lacking the A2B adenosine receptor.. J Immunol.

[39] Rork TH, Wallace KL, Kennedy DP, Marshall MA, Lankford AR (2008). Adenosine A2A receptor activation reduces infarct size in the isolated, perfused mouse heart by inhibiting resident cardiac mast cell degranulation.. Am J Physiol Heart Circ Physiol.

[40] Fenster MS, Shepherd RK, Linden J, Duling BR (2000). Activation of adenosine A2 alpha receptors inhibits mast cell degranulation and mast cell-dependent vasoconstriction.. Microcirculation.

[41] Versluis M, Postma DS, Timens W, Hylkema MN (2008). Effects of IL-4 and IL-13 on adenosine receptor expression and responsiveness of the human mast cell line 1.. Int Immunopharmacol.

